# 1:1 Co-crystal of 4,4′-(ethene-1,2-di­yl)dipyridin-1-ium sulfate and hexa­aqua­iron(II) sulfate monohydrate

**DOI:** 10.1107/S1600536814007053

**Published:** 2014-04-05

**Authors:** Dan Yang, Fei-Lin Yang

**Affiliations:** a School of Environmental and Chemical Engineering, Jiangsu University of Science and Technology, Zhenjiang 212003, People’s Republic of China

## Abstract

In the title hydrated double salt, 4,4′-(ethene-1,2-di­yl)dipyridin-1-ium hexa­aqua­iron(II) bis­(sulfate) monohydrate, (C_12_H_12_N_2_)[Fe(H_2_O)_6_](SO_4_)_2_·H_2_O, the Fe^II^ cation is coordin­ated by six water mol­ecules in a slightly distorted octa­hedral geometry; the two pyridine rings of the 4,4′-(ethene-1,2-di­yl)dipyridin-1-ium cation are twisted to each other by a dihedral angle of 11.84 (10)°. In the crystal, the cations, sulfate anions and water mol­ecules of crystallization are linked by O—H⋯O, N—H⋯O and weak C—H⋯O hydrogen bonds, forming a three-dimensional supra­molecular network.

## Related literature   

For a related structure, see: Prakash *et al.* (2012 ▶). For the synthesis, see: Bok *et al.* (1975 ▶).
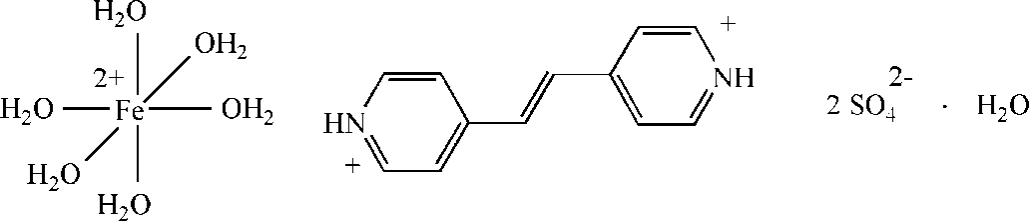


## Experimental   

### 

#### Crystal data  

(C_12_H_12_N_2_)[Fe(H_2_O)_6_](SO_4_)_2_·H_2_O
*M* *_r_* = 558.32
Triclinic, 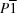

*a* = 6.772 (1) Å
*b* = 12.5006 (18) Å
*c* = 14.187 (2) Å
α = 68.991 (2)°
β = 81.829 (2)°
γ = 87.925 (2)°
*V* = 1109.6 (3) Å^3^
*Z* = 2
Mo *K*α radiation
μ = 0.94 mm^−1^
*T* = 173 K
0.26 × 0.23 × 0.08 mm

#### Data collection  

Bruker SMART APEXII diffractometer
Absorption correction: multi-scan (*SADABS*; Bruker, 2001 ▶) *T*_min_ = 0.79, *T*_max_ = 0.93
8479 measured reflections
4117 independent reflections
3601 reflections with *I* > 2σ(*I*)
*R*_int_ = 0.014

#### Refinement  

*R*[*F* ^2^ > 2σ(*F* ^2^)] = 0.027
*wR*(*F* ^2^) = 0.072
*S* = 1.04
4117 reflections
289 parameters
H-atom parameters constrained
Δρ_max_ = 0.30 e Å^−3^
Δρ_min_ = −0.42 e Å^−3^

### 

Data collection: *APEX2* (Bruker, 2007 ▶); cell refinement: *SAINT* (Bruker, 2007 ▶); data reduction: *SAINT*; program(s) used to solve structure: *SHELXS97* (Sheldrick, 2008 ▶); program(s) used to refine structure: *SHELXL97* (Sheldrick, 2008 ▶); molecular graphics: *DIAMOND* (Brandenburg, 2006 ▶); software used to prepare material for publication: *SHELXTL* (Sheldrick, 2008 ▶).

## Supplementary Material

Crystal structure: contains datablock(s) I, New_Global_Publ_Block. DOI: 10.1107/S1600536814007053/xu5782sup1.cif

Structure factors: contains datablock(s) I. DOI: 10.1107/S1600536814007053/xu5782Isup2.hkl

CCDC reference: 994508

Additional supporting information:  crystallographic information; 3D view; checkCIF report

## Figures and Tables

**Table 1:** Hydrogen-bond geometry (Å, °)

*D*—H⋯*A*	*D*—H	H⋯*A*	*D*⋯*A*	*D*—H⋯*A*
N1—H1*X*⋯O13	0.89	1.74	2.622 (2)	173
N2—H2*X*⋯O10^i^	0.89	1.75	2.631 (2)	170
O1—H1*WB*⋯O7	0.85	1.89	2.733 (2)	175
O1—H1*WA*⋯O9^ii^	0.85	1.90	2.741 (2)	170
O2—H2*WA*⋯O8	0.85	1.90	2.719 (2)	161
O2—H2*WB*⋯O12	0.85	1.86	2.711 (2)	175
O3—H3*WA*⋯O8^ii^	0.85	1.91	2.751 (2)	169
O3—H3*WB*⋯O11	0.85	1.88	2.726 (2)	174
O4—H4*WA*⋯O11^iii^	0.85	1.85	2.696 (2)	171
O4—H4*WB*⋯O12^iv^	0.85	1.86	2.710 (2)	175
O5—H5*WA*⋯O15	0.85	1.90	2.742 (2)	169
O5—H5*WB*⋯O14^iii^	0.85	1.90	2.750 (2)	175
O6—H6*WA*⋯O15^v^	0.85	1.93	2.763 (2)	165
O6—H6*WB*⋯O14^iv^	0.85	1.89	2.739 (2)	180
O15—H15*A*⋯O9^ii^	0.85	1.97	2.780 (2)	159
O15—H15*B*⋯O7^vi^	0.85	1.92	2.7628 (19)	174
C1—H1⋯O8^vii^	0.95	2.48	3.339 (3)	150
C1—H1⋯O10^vii^	0.95	2.31	3.172 (2)	150
C11—H11⋯O13^viii^	0.95	2.34	3.189 (2)	149
C11—H11⋯O14^viii^	0.95	2.43	3.291 (3)	151

Symmetry codes: (i) 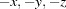; (ii) 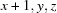; (iii) 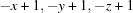; (iv) 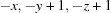; (v) 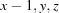; (vi) 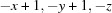; (vii) 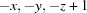; (viii) 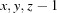.

**Fig. 1.**
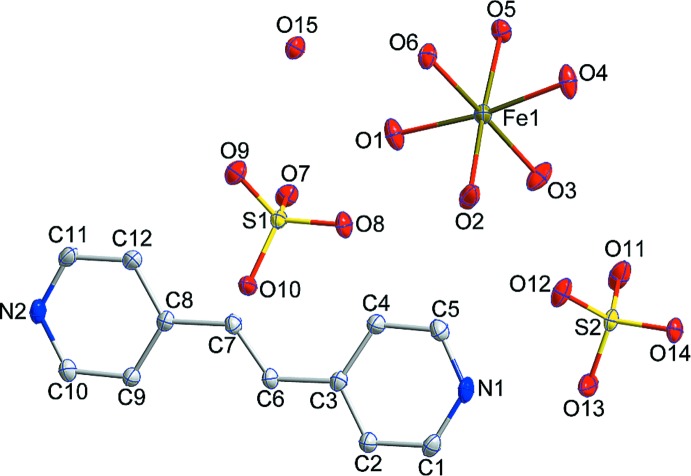
The molecular structure of the title compound with thermal ellipsoids at the 30% probability level. All H atoms were omitted for clarity.

**Fig. 2.**
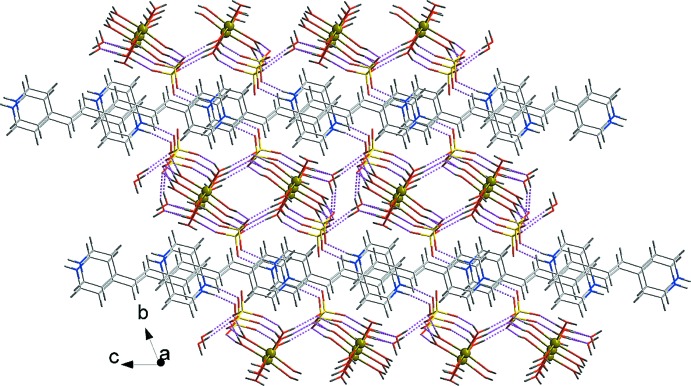
The three-dimensional supramolecular network of the title compound.

## supplementary crystallographic information

### 1. Comment

In this paper, we used [Mo(CN)_8_]^3-^ as building block to react with transition metal Fe^2+^ ions and 1,2-di(pyridin-4-yl)ethylene ligand (dpe), in order to obtain octacyanometalate-based bimetallic compound. Unfortunately, the title ion-type compound was obtained. The asymmetric unit of the title compound contains one 1,2-bis-(4-pyridyl)ethylene cation, [H_2_dpe]^2+^, two sulfate anions, one hexaaqua-iron(II) cation, and one crystallized water molecule (Fig. 1). In the structure, the Fe atom adopts a distorted slightly octahedral geometry, in which the average distance of Fe—O bonds is about 2.118 Å. The [Fe(H_2_O)_6_]^2+^ cations, sulfate anions, and guest water molecules are linked by O—H···O hydrogen bonds, forming a two-dimensional (2-D) layered structure. The N—H···O hydrogen bonds between adjacent layers generate a 3-D supramolecular network (Fig. 2). The structure of the title compound is comparable to that observed in related compound (Prakash *et al.*, 2012).

### 2. Experimental

The title compound was prepared at room temperature by slow diffusion between a CH_3_CH_2_OH/H_2_O (V/V = 2:1) solution containing FeSO_4_.7H_2_O (0.05 mmol) and dpe ligand (0.10 mmol), and a CH_3_CH_2_OH/H_2_O (V:V = 2:1) solution of [HN(*n*-C_4_H_9_)_3_]_3_[Mo(CN)_8_].4H_2_O (0.025 mmol) (Bok *et al.*, 1975). After two weeks, brown plate crystals were obtained.

### 3. Refinement

All non-H atoms were refined anisotropically. The (C)H atoms of dpe were calculated at idealized positions and included in the refinement in a riding mode. The (N)H of dpe and (O)H atoms of water molecules were located from a difference Fourier map and refined as riding [N—H = 0.89 Å, *U*(H) = 1.2*U*_eq_(N); O—H = 0.85 Å, *U*(H) = 1.5*U*_eq_(O)].

### Crystal data

**Table:**

(C_12_H_12_N_2_)[Fe(H_2_O)_6_](SO_4_)_2_·H_2_O	*Z* = 2
*M**_r_* = 558.32	*F*(000) = 580
Triclinic, *P*1	*D*_x_ = 1.671 Mg m^−^^3^
Hall symbol: -P 1	Mo *K*α radiation, λ = 0.71073 Å
*a* = 6.772 (1) Å	Cell parameters from 4117 reflections
*b* = 12.5006 (18) Å	θ = 3.0–25.6°
*c* = 14.187 (2) Å	µ = 0.94 mm^−^^1^
α = 68.991 (2)°	*T* = 173 K
β = 81.829 (2)°	Plate, brown
γ = 87.925 (2)°	0.26 × 0.23 × 0.08 mm
*V* = 1109.6 (3) Å^3^	

### Data collection

**Table:**

Bruker SMART APEXII diffractometer	4117 independent reflections
Radiation source: fine-focus sealed tube	3601 reflections with *I* > 2σ(*I*)
Graphite monochromator	*R*_int_ = 0.014
phi and ω scans	θ_max_ = 25.6°, θ_min_ = 3.0°
Absorption correction: multi-scan (*SADABS*; Bruker, 2001)	*h* = −8→8
*T*_min_ = 0.79, *T*_max_ = 0.93	*k* = −15→15
8479 measured reflections	*l* = −17→17

### Refinement

**Table:**

Refinement on *F*^2^	Primary atom site location: structure-invariant direct methods
Least-squares matrix: full	Secondary atom site location: difference Fourier map
*R*[*F*^2^ > 2σ(*F*^2^)] = 0.027	Hydrogen site location: inferred from neighbouring sites
*wR*(*F*^2^) = 0.072	H-atom parameters constrained
*S* = 1.04	*w* = 1/[σ^2^(*F*_o_^2^) + (0.0384*P*)^2^ + 0.3901*P*] where *P* = (*F*_o_^2^ + 2*F*_c_^2^)/3
4117 reflections	(Δ/σ)_max_ = 0.001
289 parameters	Δρ_max_ = 0.30 e Å^−^^3^
0 restraints	Δρ_min_ = −0.42 e Å^−^^3^

### Special details

**Table:**

Geometry. All esds (except the esd in the dihedral angle between two l.s. planes) are estimated using the full covariance matrix. The cell esds are taken into account individually in the estimation of esds in distances, angles and torsion angles; correlations between esds in cell parameters are only used when they are defined by crystal symmetry. An approximate (isotropic) treatment of cell esds is used for estimating esds involving l.s. planes.
Refinement. Refinement of *F*^2^ against ALL reflections. The weighted *R*-factor *wR* and goodness of fit *S* are based on *F*^2^, conventional *R*-factors *R* are based on *F*, with *F* set to zero for negative *F*^2^. The threshold expression of *F*^2^ > σ(*F*^2^) is used only for calculating *R*-factors(gt) *etc*. and is not relevant to the choice of reflections for refinement. *R*-factors based on *F*^2^ are statistically about twice as large as those based on *F*, and *R*- factors based on ALL data will be even larger.

### Fractional atomic coordinates and isotropic or equivalent isotropic displacement parameters (Å^2^)

**Table:**

	*x*	*y*	*z*	*U*_iso_*/*U*_eq_	
Fe1	0.30537 (4)	0.51818 (2)	0.268630 (19)	0.02402 (9)	
S1	−0.15024 (7)	0.27787 (4)	0.19605 (3)	0.02389 (11)	
S2	0.25179 (7)	0.26222 (4)	0.62987 (3)	0.02462 (11)	
N1	0.2643 (2)	0.05496 (15)	0.51392 (12)	0.0323 (4)	
H1X	0.2705	0.0772	0.5664	0.039*	
N2	0.2490 (2)	−0.07642 (15)	−0.08553 (12)	0.0319 (4)	
H2X	0.2430	−0.0967	−0.1392	0.038*	
O1	0.3515 (2)	0.42427 (13)	0.17016 (12)	0.0423 (4)	
H1WA	0.4625	0.4022	0.1478	0.063*	
H1WB	0.2660	0.3818	0.1614	0.063*	
O2	0.0864 (2)	0.40216 (14)	0.36832 (11)	0.0489 (4)	
H2WA	0.0035	0.3615	0.3555	0.073*	
H2WB	0.0820	0.3740	0.4327	0.073*	
O3	0.5165 (2)	0.42177 (14)	0.35920 (11)	0.0455 (4)	
H3WA	0.6108	0.3812	0.3446	0.068*	
H3WB	0.4920	0.3939	0.4239	0.068*	
O4	0.2662 (2)	0.63014 (14)	0.35026 (12)	0.0444 (4)	
H4WA	0.3541	0.6413	0.3831	0.067*	
H4WB	0.1579	0.6494	0.3778	0.067*	
O5	0.53157 (19)	0.63820 (11)	0.17344 (10)	0.0319 (3)	
H5WA	0.6162	0.6194	0.1321	0.048*	
H5WB	0.6023	0.6616	0.2073	0.048*	
O6	0.0777 (2)	0.61071 (12)	0.18625 (10)	0.0333 (3)	
H6WA	0.0236	0.5954	0.1419	0.050*	
H6WB	−0.0144	0.6420	0.2141	0.050*	
O7	0.0587 (2)	0.29477 (12)	0.14664 (10)	0.0339 (3)	
O8	−0.1730 (2)	0.31232 (12)	0.28575 (10)	0.0355 (3)	
O9	−0.2785 (2)	0.34627 (13)	0.12172 (11)	0.0378 (3)	
O10	−0.2059 (2)	0.15596 (11)	0.22994 (10)	0.0388 (4)	
O11	0.4357 (2)	0.31751 (15)	0.56561 (11)	0.0457 (4)	
O12	0.0825 (2)	0.29986 (14)	0.57251 (10)	0.0400 (4)	
O13	0.2703 (3)	0.13740 (12)	0.65942 (11)	0.0481 (4)	
O14	0.2198 (2)	0.28888 (12)	0.72368 (10)	0.0325 (3)	
O15	0.8392 (2)	0.57495 (12)	0.05726 (10)	0.0363 (3)	
H15A	0.8100	0.5079	0.0608	0.054*	
H15B	0.8754	0.6109	−0.0059	0.054*	
C1	0.2380 (3)	−0.05528 (18)	0.52884 (14)	0.0326 (4)	
H1	0.2182	−0.1102	0.5962	0.039*	
C2	0.2395 (3)	−0.08957 (17)	0.44725 (14)	0.0312 (4)	
H2	0.2230	−0.1684	0.4581	0.037*	
C3	0.2651 (3)	−0.00942 (16)	0.34859 (13)	0.0255 (4)	
C4	0.2926 (3)	0.10517 (17)	0.33679 (15)	0.0340 (5)	
H4	0.3117	0.1624	0.2705	0.041*	
C5	0.2919 (3)	0.13460 (18)	0.42025 (16)	0.0364 (5)	
H5	0.3112	0.2125	0.4119	0.044*	
C6	0.2634 (3)	−0.04685 (16)	0.26238 (14)	0.0303 (4)	
H6	0.2730	−0.1267	0.2750	0.036*	
C7	0.2494 (3)	0.02256 (16)	0.16764 (14)	0.0273 (4)	
H7	0.2379	0.1022	0.1554	0.033*	
C8	0.2502 (3)	−0.01417 (15)	0.08059 (13)	0.0249 (4)	
C9	0.2601 (3)	−0.12954 (16)	0.08962 (14)	0.0297 (4)	
H9	0.2673	−0.1878	0.1542	0.036*	
C10	0.2595 (3)	−0.15785 (17)	0.00524 (15)	0.0326 (4)	
H10	0.2666	−0.2360	0.0112	0.039*	
C11	0.2405 (3)	0.03440 (17)	−0.09822 (14)	0.0321 (4)	
H11	0.2340	0.0902	−0.1640	0.038*	
C12	0.2412 (3)	0.06773 (16)	−0.01616 (14)	0.0290 (4)	
H12	0.2356	0.1468	−0.0252	0.035*	

### Atomic displacement parameters (Å^2^)

**Table:**

	*U*^11^	*U*^22^	*U*^33^	*U*^12^	*U*^13^	*U*^23^
Fe1	0.02364 (15)	0.02605 (15)	0.02441 (15)	0.00119 (11)	−0.00470 (11)	−0.01102 (11)
S1	0.0272 (2)	0.0257 (2)	0.0205 (2)	−0.00155 (18)	−0.00298 (18)	−0.01036 (18)
S2	0.0258 (2)	0.0328 (2)	0.0187 (2)	0.00145 (19)	−0.00384 (17)	−0.01322 (19)
N1	0.0343 (9)	0.0449 (10)	0.0267 (9)	0.0050 (8)	−0.0070 (7)	−0.0228 (8)
N2	0.0331 (9)	0.0448 (10)	0.0233 (8)	−0.0038 (8)	−0.0019 (7)	−0.0191 (7)
O1	0.0308 (8)	0.0552 (9)	0.0579 (10)	0.0007 (7)	−0.0048 (7)	−0.0413 (8)
O2	0.0513 (10)	0.0627 (10)	0.0264 (8)	−0.0277 (8)	−0.0064 (7)	−0.0051 (7)
O3	0.0424 (9)	0.0622 (10)	0.0300 (8)	0.0226 (8)	−0.0101 (7)	−0.0144 (7)
O4	0.0270 (8)	0.0698 (11)	0.0579 (10)	0.0054 (7)	−0.0072 (7)	−0.0486 (9)
O5	0.0273 (7)	0.0379 (8)	0.0338 (7)	−0.0032 (6)	−0.0007 (6)	−0.0177 (6)
O6	0.0294 (7)	0.0426 (8)	0.0345 (8)	0.0093 (6)	−0.0119 (6)	−0.0198 (6)
O7	0.0279 (7)	0.0415 (8)	0.0301 (7)	0.0005 (6)	−0.0014 (6)	−0.0111 (6)
O8	0.0384 (8)	0.0436 (8)	0.0344 (8)	0.0005 (6)	−0.0042 (6)	−0.0263 (7)
O9	0.0337 (8)	0.0450 (9)	0.0342 (8)	0.0066 (6)	−0.0112 (6)	−0.0118 (7)
O10	0.0636 (10)	0.0299 (7)	0.0232 (7)	−0.0138 (7)	−0.0015 (7)	−0.0104 (6)
O11	0.0299 (8)	0.0764 (12)	0.0274 (8)	−0.0122 (8)	0.0002 (6)	−0.0150 (8)
O12	0.0278 (8)	0.0661 (10)	0.0281 (7)	0.0064 (7)	−0.0081 (6)	−0.0181 (7)
O13	0.0878 (13)	0.0342 (8)	0.0298 (8)	0.0099 (8)	−0.0152 (8)	−0.0185 (7)
O14	0.0366 (8)	0.0414 (8)	0.0274 (7)	0.0038 (6)	−0.0050 (6)	−0.0218 (6)
O15	0.0439 (9)	0.0407 (8)	0.0249 (7)	−0.0010 (7)	−0.0013 (6)	−0.0137 (6)
C1	0.0354 (11)	0.0403 (11)	0.0201 (9)	−0.0005 (9)	−0.0024 (8)	−0.0088 (8)
C2	0.0416 (12)	0.0284 (10)	0.0250 (10)	0.0002 (9)	−0.0062 (8)	−0.0105 (8)
C3	0.0270 (10)	0.0288 (10)	0.0227 (9)	0.0022 (8)	−0.0042 (7)	−0.0114 (8)
C4	0.0509 (13)	0.0268 (10)	0.0251 (10)	−0.0001 (9)	−0.0075 (9)	−0.0093 (8)
C5	0.0487 (13)	0.0309 (11)	0.0361 (11)	0.0030 (9)	−0.0109 (10)	−0.0182 (9)
C6	0.0434 (12)	0.0269 (10)	0.0242 (10)	0.0004 (8)	−0.0047 (8)	−0.0134 (8)
C7	0.0336 (11)	0.0266 (9)	0.0260 (10)	0.0036 (8)	−0.0072 (8)	−0.0139 (8)
C8	0.0255 (10)	0.0285 (9)	0.0218 (9)	0.0008 (8)	−0.0043 (7)	−0.0101 (8)
C9	0.0413 (12)	0.0267 (10)	0.0223 (9)	0.0018 (8)	−0.0075 (8)	−0.0091 (8)
C10	0.0404 (12)	0.0314 (10)	0.0302 (10)	−0.0005 (9)	−0.0045 (9)	−0.0163 (9)
C11	0.0355 (11)	0.0372 (11)	0.0195 (9)	−0.0052 (9)	−0.0039 (8)	−0.0046 (8)
C12	0.0337 (11)	0.0281 (10)	0.0246 (9)	−0.0025 (8)	−0.0054 (8)	−0.0079 (8)

### Geometric parameters (Å, º)

**Table:**

Fe1—O2	2.0954 (14)	O5—H5WA	0.8500
Fe1—O4	2.1019 (14)	O5—H5WB	0.8500
Fe1—O3	2.1059 (14)	O6—H6WA	0.8502
Fe1—O1	2.1101 (14)	O6—H6WB	0.8498
Fe1—O6	2.1199 (13)	O15—H15A	0.8499
Fe1—O5	2.1323 (13)	O15—H15B	0.8498
S1—O9	1.4690 (14)	C1—C2	1.370 (3)
S1—O10	1.4698 (14)	C1—H1	0.9500
S1—O8	1.4700 (13)	C2—C3	1.390 (3)
S1—O7	1.4738 (14)	C2—H2	0.9500
S2—O11	1.4656 (15)	C3—C4	1.397 (3)
S2—O12	1.4663 (14)	C3—C6	1.459 (2)
S2—O13	1.4690 (15)	C4—C5	1.359 (3)
S2—O14	1.4696 (13)	C4—H4	0.9500
N1—C1	1.332 (3)	C5—H5	0.9500
N1—C5	1.336 (3)	C6—C7	1.328 (3)
N1—H1X	0.8902	C6—H6	0.9500
N2—C11	1.332 (3)	C7—C8	1.463 (2)
N2—C10	1.334 (3)	C7—H7	0.9500
N2—H2X	0.8901	C8—C12	1.396 (3)
O1—H1WA	0.8500	C8—C9	1.401 (3)
O1—H1WB	0.8500	C9—C10	1.365 (2)
O2—H2WA	0.8499	C9—H9	0.9500
O2—H2WB	0.8499	C10—H10	0.9500
O3—H3WA	0.8499	C11—C12	1.371 (3)
O3—H3WB	0.8506	C11—H11	0.9500
O4—H4WA	0.8500	C12—H12	0.9500
O4—H4WB	0.8500		
			
O2—Fe1—O4	93.26 (6)	H4WA—O4—H4WB	104.0
O2—Fe1—O3	87.76 (6)	Fe1—O5—H5WA	119.6
O4—Fe1—O3	92.10 (6)	Fe1—O5—H5WB	112.5
O2—Fe1—O1	92.61 (6)	H5WA—O5—H5WB	104.2
O4—Fe1—O1	172.85 (6)	Fe1—O6—H6WA	127.3
O3—Fe1—O1	92.22 (6)	Fe1—O6—H6WB	120.8
O2—Fe1—O6	88.65 (6)	H6WA—O6—H6WB	104.1
O4—Fe1—O6	86.54 (6)	H15A—O15—H15B	104.2
O3—Fe1—O6	176.09 (5)	N1—C1—C2	120.11 (18)
O1—Fe1—O6	89.51 (6)	N1—C1—H1	119.9
O2—Fe1—O5	177.16 (5)	C2—C1—H1	119.9
O4—Fe1—O5	84.35 (6)	C1—C2—C3	120.25 (18)
O3—Fe1—O5	90.80 (6)	C1—C2—H2	119.9
O1—Fe1—O5	89.89 (6)	C3—C2—H2	119.9
O6—Fe1—O5	92.72 (5)	C2—C3—C4	117.53 (16)
O9—S1—O10	109.65 (9)	C2—C3—C6	119.71 (16)
O9—S1—O8	110.74 (9)	C4—C3—C6	122.76 (17)
O10—S1—O8	108.44 (8)	C5—C4—C3	119.92 (18)
O9—S1—O7	108.66 (8)	C5—C4—H4	120.0
O10—S1—O7	109.39 (9)	C3—C4—H4	120.0
O8—S1—O7	109.95 (8)	N1—C5—C4	120.65 (18)
O11—S2—O12	109.56 (9)	N1—C5—H5	119.7
O11—S2—O13	108.96 (10)	C4—C5—H5	119.7
O12—S2—O13	109.22 (9)	C7—C6—C3	124.73 (17)
O11—S2—O14	110.64 (8)	C7—C6—H6	117.6
O12—S2—O14	110.64 (8)	C3—C6—H6	117.6
O13—S2—O14	107.78 (8)	C6—C7—C8	125.04 (17)
C1—N1—C5	121.54 (16)	C6—C7—H7	117.5
C1—N1—H1X	120.5	C8—C7—H7	117.5
C5—N1—H1X	117.9	C12—C8—C9	117.67 (16)
C11—N2—C10	122.22 (16)	C12—C8—C7	119.59 (16)
C11—N2—H2X	118.6	C9—C8—C7	122.74 (16)
C10—N2—H2X	119.1	C10—C9—C8	119.68 (18)
Fe1—O1—H1WA	126.9	C10—C9—H9	120.2
Fe1—O1—H1WB	126.3	C8—C9—H9	120.2
H1WA—O1—H1WB	103.8	N2—C10—C9	120.40 (18)
Fe1—O2—H2WA	129.5	N2—C10—H10	119.8
Fe1—O2—H2WB	124.8	C9—C10—H10	119.8
H2WA—O2—H2WB	104.3	N2—C11—C12	119.85 (17)
Fe1—O3—H3WA	129.3	N2—C11—H11	120.1
Fe1—O3—H3WB	122.0	C12—C11—H11	120.1
H3WA—O3—H3WB	104.3	C11—C12—C8	120.17 (18)
Fe1—O4—H4WA	123.2	C11—C12—H12	119.9
Fe1—O4—H4WB	128.2	C8—C12—H12	119.9

### Hydrogen-bond geometry (Å, º)

**Table:**

*D*—H···*A*	*D*—H	H···*A*	*D*···*A*	*D*—H···*A*
N1—H1*X*···O13	0.89	1.74	2.622 (2)	173
N2—H2*X*···O10^i^	0.89	1.75	2.631 (2)	170
O1—H1*WB*···O7	0.85	1.89	2.733 (2)	175
O1—H1*WA*···O9^ii^	0.85	1.90	2.741 (2)	170
O2—H2*WA*···O8	0.85	1.90	2.719 (2)	161
O2—H2*WB*···O12	0.85	1.86	2.711 (2)	175
O3—H3*WA*···O8^ii^	0.85	1.91	2.751 (2)	169
O3—H3*WB*···O11	0.85	1.88	2.726 (2)	174
O4—H4*WA*···O11^iii^	0.85	1.85	2.696 (2)	171
O4—H4*WB*···O12^iv^	0.85	1.86	2.710 (2)	175
O5—H5*WA*···O15	0.85	1.90	2.742 (2)	169
O5—H5*WB*···O14^iii^	0.85	1.90	2.750 (2)	175
O6—H6*WA*···O15^v^	0.85	1.93	2.763 (2)	165
O6—H6*WB*···O14^iv^	0.85	1.89	2.739 (2)	180
O15—H15*A*···O9^ii^	0.85	1.97	2.780 (2)	159
O15—H15*B*···O7^vi^	0.85	1.92	2.7628 (19)	174
C1—H1···O8^vii^	0.95	2.48	3.339 (3)	150
C1—H1···O10^vii^	0.95	2.31	3.172 (2)	150
C11—H11···O13^viii^	0.95	2.34	3.189 (2)	149
C11—H11···O14^viii^	0.95	2.43	3.291 (3)	151

Symmetry codes: (i) −*x*, −*y*, −*z*; (ii) *x*+1, *y*, *z*; (iii) −*x*+1, −*y*+1, −*z*+1; (iv) −*x*, −*y*+1, −*z*+1; (v) *x*−1, *y*, *z*; (vi) −*x*+1, −*y*+1, −*z*; (vii) −*x*, −*y*, −*z*+1; (viii) *x*, *y*, *z*−1.
